# Establishment of the multi-component bone-on-a-chip: to explore therapeutic potential of DNA aptamers on endothelial cells

**DOI:** 10.3389/fcell.2023.1183163

**Published:** 2023-06-12

**Authors:** Xiaoyu Fan, Yuhan Yan, Lianhui Zhao, Xin Xu, Yiyang Dong, Wei Sun

**Affiliations:** ^1^ Peking University Health Science Center, China-Japan Friendship School of Clinical Medicine, Beijing, China; ^2^ Department of Pharmacy, College of Life Science and Technology, Beijing University of Chemical Technology, Beijing, China; ^3^ Peking Union Medical College, China-Japan Friendship School of Clinical Medicine, Beijing, China; ^4^ Orthopedics Department, China-Japan Friendship Hospital, Beijing, China; ^5^ Department of Orthopaedic Surgery, Perelman School of Medicine, University of Pennsylvania, Philadelphia, PA, United States

**Keywords:** bone-on-a-chip, microfluidic system, osteonecrosis of the femoral head, aptamer, multi-component

## Abstract

**Background:** Despite great efforts to develop microvascular bone chips in previous studies, current bone chips still lacked multi-component of human-derived cells close to human bone tissue. Bone microvascular endothelial cells (BMECs) were demonstrated to be closely related to the glucocorticoid (GC)-induced osteonecrosis of the femoral head (ONFH). Tumor necrosis factor-alpha (TNF-α) aptamer has been proved to bind to its receptor and block cascade activities.

**Objective:** There are two main objectives in this study: 1) to establish a multi-component bone-on-a-chip within the microfluidic system *in vitro*, 2) to explore the therapeutic potential of TNF-α aptamer on BMECs in the GC-induced ONFH model.

**Methods:** Histological features of clinical samples were analyzed before BMECs isolation. The functional bone-on-a-chip consists of the vascular channel, stromal channel and structure channel. GC-induced ONFH model was established based on the multi-component of human-derived cells. Truncation and dimerization were performed on a previously reported DNA aptamer (VR11). BMECs apoptosis, cytoskeleton and angiogenesis status in the ONFH model were observed by the TUNEL staining and confocal microscope.

**Results:** The multi-component of BMECs, human embryonic lung fibroblasts and hydroxyapatite were cultured within the microfluidic bone-on-a-chip. TNF-α was found up-regulated in the necrotic regions of femoral heads in clinical samples and similar results were re-confirmed in the ONFH model established in the microfluidic platform by detecting cell metabolites. Molecular docking simulations indicated that the truncated TNF-α aptamer could improve the aptamer-protein interactions. Further results from the TUNEL staining and confocal microscopy showed that the truncated aptamer could protect BMECs from apoptosis and alleviate GC-induced damages to cytoskeleton and vascularization.

**Conclusion:** In summary, a microfluidic multi-component bone-on-a-chip was established with ‘off-chip’ analysis of cell metabolism. GC-induced ONFH model was achieved based on the platform. Our findings provided initial evidence on the possible potentials of TNF-α aptamer as a new type of TNF-α inhibitor for patients with ONFH.

## 1 Introduction

The injury of blood supply and angiogenesis to the tissue is considered as the core of osteonecrosis of the femoral head (ONFH) (Petek et al., 2019). Exposure to a long-term glucocorticoids intake has been proved to be highly related to the onset and development of ONFH (Kaneko et al., 2021; Krez et al., 2021; Weinstein, 2012). Previous studies demonstrated that the impairments of bone microvascular endothelial cells (BMECs) by dexamethasone might play an important role in the glucocorticoid-induced osteonecrosis (Wu et al., 2022). Researchers found that communications between endothelial cells and fibroblasts were improtant in maintaining cell morphologies and avoiding regressions of the microvascular networks (Whisler et al., 2014). The newly-formed microvascular networks can also be stabilized by the recruited fibroblasts, which mainly contribute to the deposition of local extracellular matrix (Jeon et al., 2014).

However, conventional cell culture devices can not fully support the needs for the tissue-specific interactions or differentiated functions of various cells in ONFH model. On multiple occasions, animal models could be unsuccessful and unreliable in developing the human bone pathologies and predicting responses to drug candidates, because their physiology is fundamentally different from that of humans. Organ-on-a-chip is a microfluidic device which is capable of culturing multiple cell types within continuously perfused, micrometersized chambers in order to mimic physiological functions of *in vivo* tissues and organs by combining the advances in cell engineering with microfluidic technology (Kim et al., 2013). By far, several studies have provided sufficient experimental evidence of the superiority and feasibility of bone chips compared with traditional devices of *in vitro* cell culture (Chou et al., 2020; Glaser et al., 2022; Jusoh et al., 2015; Li et al., 2021). Unfortunately, these facilities either lacked multi-component of human-derived cells close to human bone tissue or had inadequate ‘off-chip’ analysis of cell metabolism from the devices.

Generally, endothelial cells express a broad spectrum of membrane receptors, such as the receptors of tumor necrosis factor-alpha (TNF-α), vascular endothelial growth factor (VEGF) family and etc (Aggarwal et al., 2012). Evidence was found that the infiltrating inflammatory macrophages, through their expression of TNF-α, could directly induce apoptosis of the epithelial cells and fibro/adipogenic progenitors (Gunther et al., 2011; Lemos et al., 2015). Recently, TNF-α-induced cell death has been confirmed in the early development of ONFH and TNF-α inhibitors was used to alleviate this damage to bone tissues (Ji et al., 2019; Zheng et al., 2020). An aptamer is a single-stranded DNA or RNA oligonucleotide sequence, similar to monoclonal antibody, which can bind to a wide range of receptors with high specificity and affinity. A previously reported DNA aptamer (VR11) was proved to bind to TNF-α with a high affinity and block its activities *in vitro* (Orava et al., 2013). Compared to their monoclonal antibody counterparts, aptamers are thermo-stable and easily modified. Despite the small molecular weight of aptamers, it is typical that only certain domains within the sequence are essential for its major function. Therefore, in order to improve its binding affinitiy or stability, rational truncations or dimerizations of aptamers have become commonly used methods (Hassan et al., 2021; Ohuchi and Suess, 2018).

To date, there is no study that explores the therapeutic potential of DNA aptamers in ONFH model based on bone-on-a-chip. Here we present a robust microfluidic platform which is capable of culturing multi-component of BMECs, human lung fibroblasts (HLFs) and hydroxyapatite (HA). There are two main objectives in this study: 1) to establish a multi-component ONFH model based on microfluidic system *in vitro*, 2) to explore the therapeutic potential of DNA aptamers on BMECs in the glucocorticoid (GC)-induced ONFH model.

## 2 Materials and methods

### 2.1 Patients selection

The bone tissue was obtained from the patients who were hospitalized in our orthopedic department between January 2022 and March 2022. A total of 20 samples of femoral heads were collected from 10 ONFH patients and 10 control patients (femoral neck fracture). All patients received unilateral total hip arthroplasty (THA). The deatiled characteristics of all enrolled patients are shown in the Supplementary Table S1. We selected the patients diagnosed with femoral neck fracture as the control group for two reasons: 1) it was not proper to choose patients diagnosed with osteoarthritis or rheumatoid arthritis as the control group because of we aimed to study the expression of an inflammatory biomarkers (TNF-α) in clinical samples. 2) we would extract BMECs from the clinical samples of the control group for subsequent cell experiments. Therefore, patients without any previous bone disorders (except osteoporosis) were expected in the control group. There was no significant difference between the ONFH and fracture groups with regard to sex and body mass index. Informed consent documents for femoral head donation were obtained from all enrolled patients, and all the experiments on human samples in our study were approved by the medical ethics committee of the China-Japan Friendship Hospital.

### 2.2 Haematoxylin and eosin (HE) staining and immunohistochemistry

The collected bone tissues were fixed in 10% formalin for 24 h, after which they were washed with 0.9% saline three times. Then, all samples were decalcified in 10% EDTA decalcifying solution for 3 months. All paraffin slices were subsequently dehydrated in xylene and various concentrations of ethanol several times before staining with haematoxylin and eosin. After dehydration and sealing, each slice was observed using an electron microscope (LEICA Microsystem, Germany) to examine the necrotic area and its surrounding tissues.

Similar to the first step in HE staining, dehydration was followed by the repair of protease K. The slides were blocked with 3% BSA for 30 min after being washed with PBS three times. The reaction liquid in the TUNEL kit was added to each slide before incubation with primary antibodies and secondary antibodies. The whole processes of HE staining and immunofluorescence staining were previously reported elsewhere (Yu et al., 2020). A fluorescence microscope (Olympus, Tokyo, Japan) was used to observe the immunohistochemistry images after sealing with antifade solution. The imaging data were extracted and analyzed by the ImageScope and ImageJ software. Each group consisted of three repeated slices. For each staining method, the two groups took samples from similar locations.

### 2.3 Isolation and characterization of BMECs

BMECs were extracted from the sterile samples of femoral heads in the control group according to previous protocols (Li et al., 2021). Briefly, the bone debris was first washed with Dulbecco’s modified Eagle’s medium (DMEM, Gibco, USA) three times and then digested with 0.2% type I collagenase for 25 min and 0.25% trypsin-EDTA for 5 min at 37°C. The digestion process was terminated with the addition of DMEM containing 10% fetal bovine serum (FBS). Then, the cell mixture was filtered by a 70 μm cell strainer and centrifuged at 800 rpm for 6 min. Finally, the supernates was removed, and the cells in the deposits were resuspended in 5 ml endothelial cell medium (ECM, ScienCell, USA) for cell counting. BMECs were then cultured in ECM (Gibco BRL, Life Technologies) containing 10% fetal bovine serum, 1% recombinant human vascular endothelial growth factor (VEGF) and 1% streptomycin-penicillin solution in a 37°C humidified incubator with 5% CO_2_.

Similar to the BMEC characterization reported previously (Wu et al., 2022). CD31 and vWF were selected as the characteristic markers of human BMEC. Immunofluorescence staining of the BMECs in 75 ml culture flask were applied for BMEC characterization. After being fixed by 4% paraformaldehyde and blocked by 5% BSA, BMECs were incubated with mouse antibodies against CD31 (CST, 1:1000) and vWF (CST, 1:1000) at 4°C overnight. Then the Anti-rat IgG (H+L) (Alexa Fluor^®^ 488 Conjugate) secondary antibody (CST, 1:2000) were incubated at 37°C for 30 min on the second day. The DAPI and merged images were captured by a Fluorescence Microscope (Casse, USA). The imaging data were extracted and analyzed by ImageJ software (https://imagej.nih.gov/ij/). Each staining contains three independent wells in each plate.

### 2.4 Device fabrication and assembly

The bone-on-a-chip was designed and fabricated by our joint group, and the process of device assembling was reported as follows. The mold was manufactured by SU-8 photolithography method. Silane treatment was performed after the liquid polydimethylsiloxane (PDMS; Sylgard Dow Corning) (the ratio of prepolymer to curing agent was 10:1) was poured on the positive mold, followed by degassing and after curing at vacuum drying oven for 30 min. The holes with a diameter of 100 μm were punched at the positions of the injection sites, followed by dust-free treatment of all chips. The glass plate and the chip were placed in a plastic petri dish with the structural side facing upwards in order to complete plasma bonding. The petri dish was placed in an oxygen cleaner, and after vacuuming for 1 min, air was injected to attach the structural side of the chip, and the chip was placed flat for 12 h. All chips were sterilized by 75% ethanol (12 h) and ultraviolet light (2 h).

### 2.5 Multi-component culture of BMECs, human lung fibroblasts (HLFs) and hydroxyapatite (HA)

The isolation and characterization of BMECs were mentioned above. The HLF was provided by National Infrastructure of Cell Line Resource (NICR). The fibroblasts were cultured in 0.9% Glu-DMEM (GibcoBRL, Life Technologies) containing 10% fetal bovine serum in a 37°C humidified incubator with 5% CO2. The BMECs or HLFs were passaged or harvested for subsequent experiments at the confluency of 70%–80%.

Furthermore, three groups with different culture patterns were designed for evaluation: Group I (BMECs, the control group), the green channel was filled with mixture of rat matrix collagen gel, ECM and BMECs suspension (1.0*10^6^ cells/ml). The yellow channel was filled with rat collagen gel only. Group II (BMECs+0.2% HA, the two-component group), the green channel was filled with mixture of rat matrix collagen gel, 0.2% HA, ECM and BMECs suspension (1.0*10^6^ cells/ml). The yellow channel was filled with rat collagen gel only. Group III (BMEC+0.2%HA+HLF, the multi-component group), the green channel was filled with mixture of rat matrix collagen gel, 0.2% HA, ECM and BMECs suspension (1.0*10^6^ cells/ml). The yellow channel was filled with the mixture of HLFs suspension (5*105 cells/mL), MEM (containing 10% FBS) and rat matrix collagen gel. In all three groups, the purple channel was used for ECM reservation and the blue channel was filled with rat collagen gel with 0.2% HA as the structure channel. The detailed components of different culture patterns are summarized in the Supplementary Table S2.

### 2.6 Collection of cell metabolites in microfluidic system

After the establishment of the multi-component model, the inlets and outlets of the channels were blocked by the polydimethylsiloxane (PDMS; Sylgard Dow Corning) except for a pair of sites in the top (purple) channel. The remaining inlet was connected to a microinfusion pump while the outlet was connected to a syringe to collect the effluent. To build the GC-induced ONFH model, the BMECs were treated with a concentration gradient of dexamethasone (DEX, Shanghai, China) through the microinfusion pump. To explore the optimal DEX condition in the microfluidic system, a gradient of 200μM, 300μM, 400μM and 500 μM DEX was applied to the multi-component model, respectively. The speed of the infusion was set at 10 μl/h with 24-h continuous flow without affecting the growth of cells in the adjacent channels. Cell metabolites from the microfluidic system was collected from the outlet. The effluent from three chips under the same DEX concentration was collected simultaneously.

### 2.7 Detection of TNF-α in cell metabolites

The concentrations of TNF-α after the interventions in the cell metabolites were quantified by the enzyme-linked immunosorbent assay (ELISA). The supernates collected from the microfluidic system was centrifuged at 1000x rpm at 4°C for 5 min. All reagents and standard dilutions were prepared fresh before adding samples to the precoated plate (Solarbio, Beijing,China). Each well was incubated with 100 μl of standard dilution or sample at 37°C for 90 min. Then 100 μl of working solution of Biotin-Conjugate anti-human TNF-α antibody was added to each well for 60 min after plate washing. Similar procedures were performed with the addition of 100 μl working solutions of Streptavidin-HRP to each well for another 30 min. All wells were then aspirated and washed for 5 times, followed by the addition of 100 μl substrate solutions away from light. Another 50 μl of stop solution was added to each well before measuring absorbance values at 450 nm in the microplate reader (EL800,Bio-Tek Instrument,USA). The reference wavelength was set at 630 nm. The whole process of ELISA was previously reported elsewhere (Porsbjerg et al., 2021).

### 2.8 Model simulation of aptamer docking with TNF-α

The sequence of original aptamer ‘VR11’ is 5′-TGG​TGG​ATG​GCG​CAG​TCG​GCG ACAA-3’. The secondary structure of DNA aptamer (VR11) was predicted using the Mfold (http://www.unafold.org/mfold/applications/dna-folding-form.php) and the tertiary structure were predicted using the software RNA Composer (http://rnacomposer.ibch.poznan.pl/). Based on the predicted secondary structure of the original aptamer, the modified aptamers were constructed in the conceptions of truncation and mutation with a more stable stem-loop structure to acquire better affinity and suitability as expected. Docking results of the modified aptamers were analyzed using the MOE 2020 software, a program which was previously reported (Shin et al., 2020), taking the binding energy and score of clusters into full considerations. The docking analysis of the protein-aptamer interactions between TNF-α and the modified aptamers was performed before further application.

### 2.9 Inhibitory effect of modified aptamer on cell apoptosis

Based on the multi-component chips, four groups were designed in this part: group I (the control group), group II (the DEX group), group III (the DEX + VR11 group) and group IV (the DEX + modified VR11 group), each group consisting of three chips. According to the results of docking analysis, the optimally modified aptamer was selected and synthesized *in vitro*. The synthesized aptamer was centrifuged in 4000 rpm for 60s before decapping, followed by the dissolution in ECM. The dissolved aptamer was exposed to 95°C metal bath for 3 min and recooled in 4°C for 10 min before mixing with ECM (at a final concentration of 2 μM), following the injection of 10 μl ECM containing the DEX. The inhibitory effect of the modified aptamer and VR11 on cell apoptosis was assessed by the TUNEL assay after 24-h treatment. All procedures of the TUNEL assay were previously reported elsewhere (Yu et al., 2020).

### 2.10 Confocal microscopy

To observe the status of cytoskeleton and angiogenesis within the chips, the medium was aspirated and the chips were washed with PBS, followed by fixation with 4% formaldehyde and treatment with 0.3% Triton X-100. Then cells were stained with DAPI (364 nm, Sigma-Aldrich; Merck KGaA) and FITC-phalloidin (496 nm, Invitrogen; Thermo Fisher Scientific, Inc.) in a proper sequence. All images were captured by the confocal microscope (LEICA, Germany) using the 10-fold, 20-fold and 40-fold lens. The interface with optimal cell morphology, clear staining and no bubble interference was selected for final analysis. The ratio of microvessel area (capillary-like lumen area) to the whole cytoskeleton area is defined as the effective vascularization ratio to evaluate the status of angiogenesis.

### 2.11 Statistical analysis

The coverage of cytoskeleton area, effective vascularization ratio and the percent of cells positive for TUNEL assay were recorded in the form of mean ± standard deviation (SD). Sample size (n) for each analysis was elucidated in the corresponding figure legend. Data analysis was performed on SPSS Statistics 25 (Chicago, IL, USA). For the comparison between two groups, the Student’t-test or the Chi-square test was used. For comparisons between multiple groups, one-way repeated-measures ANOVA was used, followed by the LSD multiple comparison test. The imaging data were extracted and analyzed by ImageJ software (https://imagej.nih.gov/ij/). A value of *p* < 0.05 was considered to be statistically significant.

## 3 Results

### 3.1 Histological features and characterization of BMECs

**FIGURE 1:**
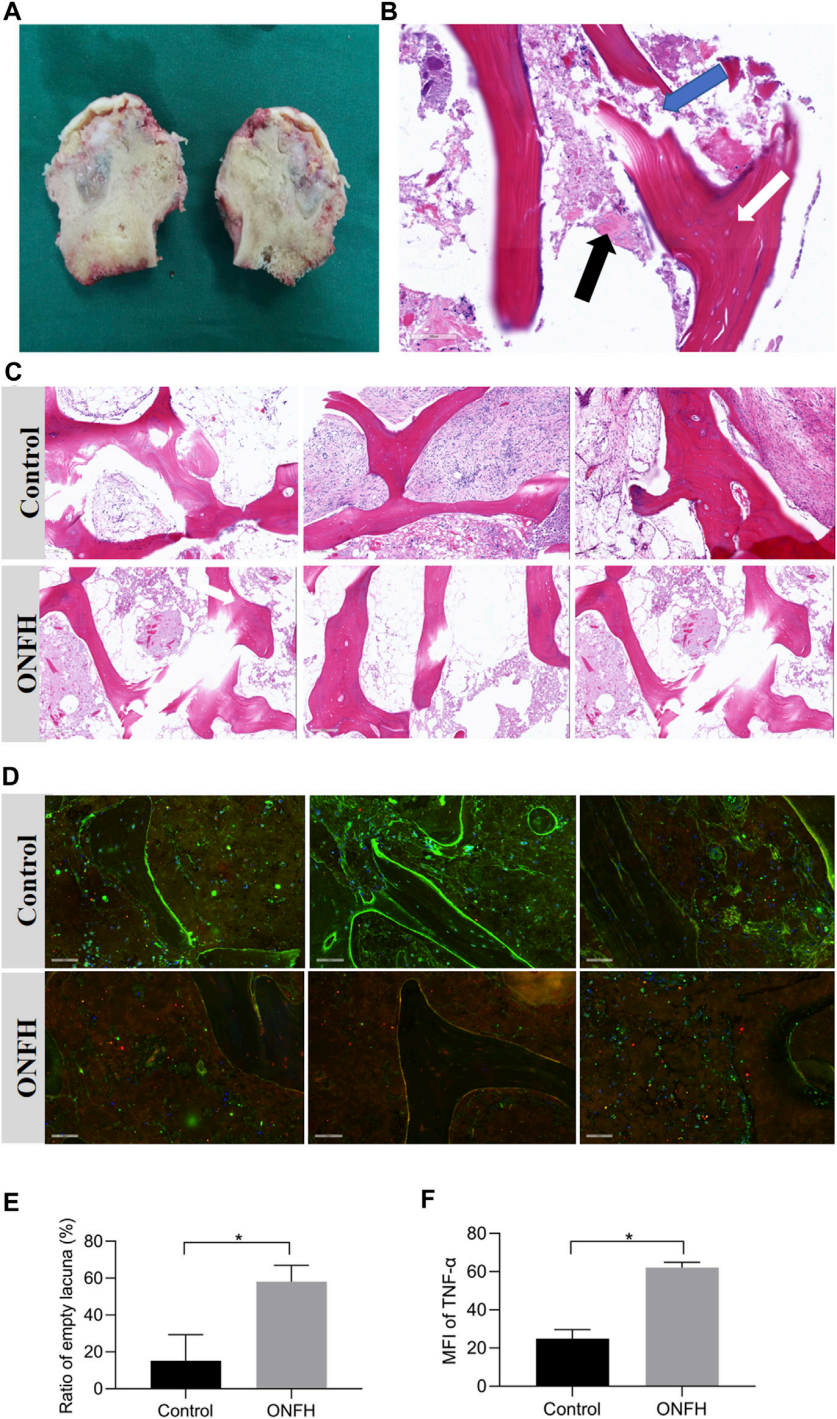
Clinical samples of femoral heads in ONFH group and histological analysis. **(A)**, clinical samples of the necrotic femoral head in ONFH patients. **(B)**, a vertical section of the necrotic femoral head in HE staining. The existence of empty lacuna (white arrow), amorphous substance (black arrow) and trabecular fracture (blue arrow) are indicated. **(C)**, the histological comparison between the control and ONFH groups in HE staining. **(D)**, the immunohistochemistry staining results of the control and ONFH groups. **(E)**, the ratio of empty lacuna in the control and ONFH groups (n = 3, each group consisted of three repeated slices). **(F)**, The mean fluorescence intensity (MFI) of TNF-α (red) in the control and ONFH groups (n = 3, each group consisted of three repeated slices). *p < 0.05. Error bars indicate SD. ONFH, osteonecrosis of the femoral head.

The clinical samples of the femoral head in the ONFH group and the HE staining are illustrated in Figures 1A, B. The thickness of the each slice was 4 μm. And there were 5 slices for each patient in total, including 1 unstained slice and 1 slice for pre-experiments. The HE staining images showed empty lacuna, amorphous substances and adipocyte infiltration in the ONFH patients. In addition, trabecular fractures could be observed in the subchondral region of the patients in the ONFH group, while normal trabecular structure and luxuriant osteocytes were observed in the control group (Figure 1C). The higher content of adipocytes in the bone marrow spaces in the ONFH group also deteriorated mechanical properties for bone tissue. The ratio of empty lacuna between the two groups was quantified in the Figure 1E.

The mean fluorescence intensity (MFI) was applied to quantify the expression levels of biological targets in immunohistochemistry images. The expression of TNF-α (red) in bone tissue of two groups (ONFH and control group) was illustrated and quantified in the Figures 1D, F. According to the analysis of the immunohistochemistry images, the MFI of TNF-α in the necrotic regions of the ONFH group was significantly higher than that in the normal regions of the control group. These histological features indicated the solid changes in the necrotic bone tissue and the potential correlation of TNF-α with the development of ONFH.

**FIGURE 2:**
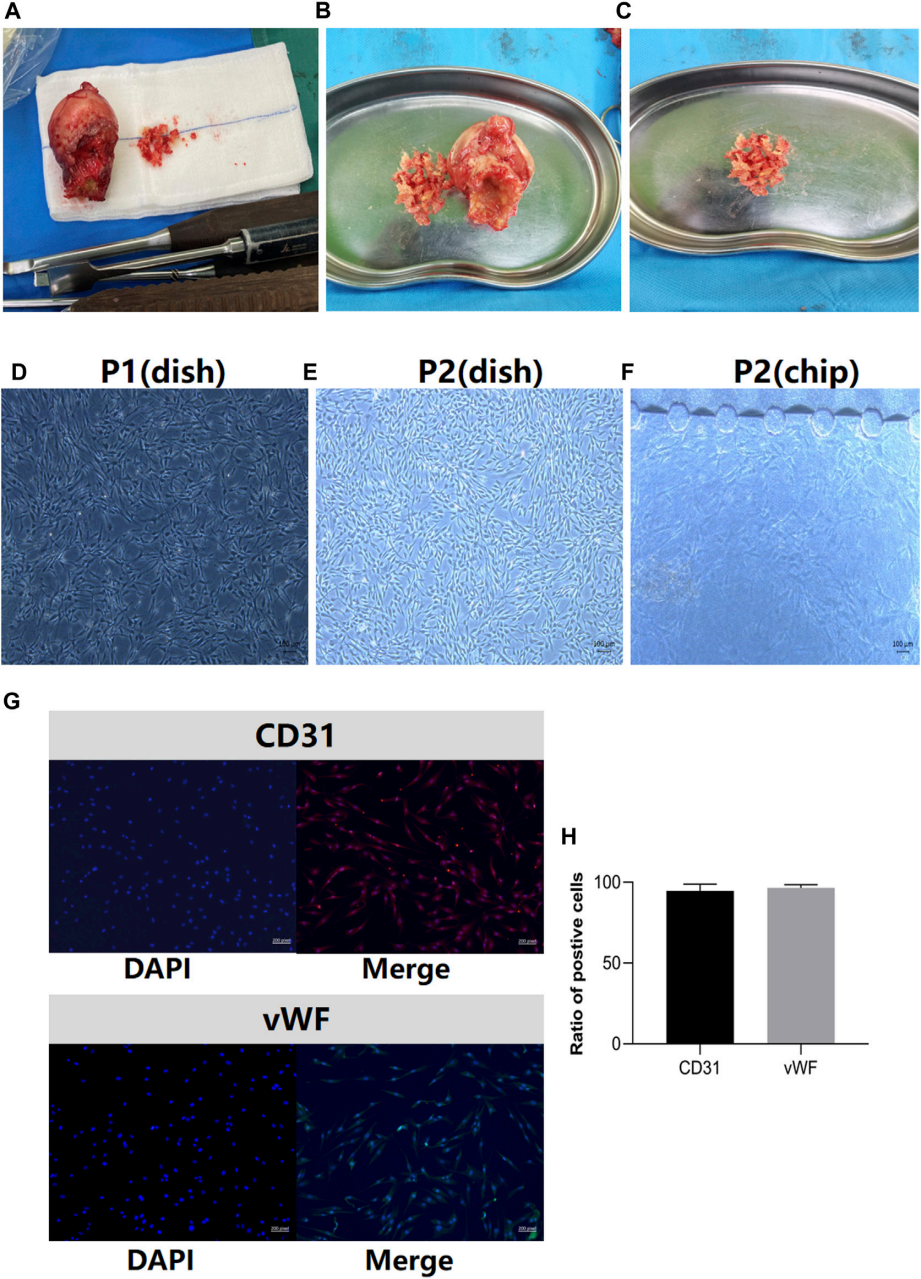
Clinical samples of femoral heads in the control group and BMECs characterization. **(A)**, clinical samples of the femoral head after in the control group. **(B)**, the process of bone extraction from the region of head-neck junction. **(C)**, the bone debris washed by the normal saline for BMECs isolation. **(D)**, the first passage (P1) of the BMECs in petri dish. **(E)**, the second passage (P2) of BMECs in petri dish. **(F)**, the features of P2 BMECs seeded in the bone-on-a-chip. **(G)**, BMECs characterization by the marker of CD31 and vWF. Left panel stands for the DAPI image, right panel stands for the merged image for each marker. **(H)**, the ratio of positive cells in CD31 and vWF staining (n = 3, each group consisted of three repeated wells). Error bars indicate SD. BMEC, bone microvascular endothelial cell.

The clinical samples of the sterile femoral head and bone debris in the control group for BMEC isolation are illustrated in Figures 2A–C. The characterization of BMECs was verified on the first passage of the cells in petri dish (Figure 2D) and all following experiments were performed on the second passages (P2) of the cells (Figure 2E). Similar to the cell morphology in petri dish, the P2 BMECs exhibited short-spindle, polygon shaped and cobblestone-like features when cultured in bone-on-a-chip (Figure 2F). High expressions of CD31 and vWF in BMECs were confirmed before cell seeding in the bone-on-a-chip (Figures 2G, H).

### 3.2 Bone-on-a-chip fabrication and microfluidic devices

**FIGURE 3:**
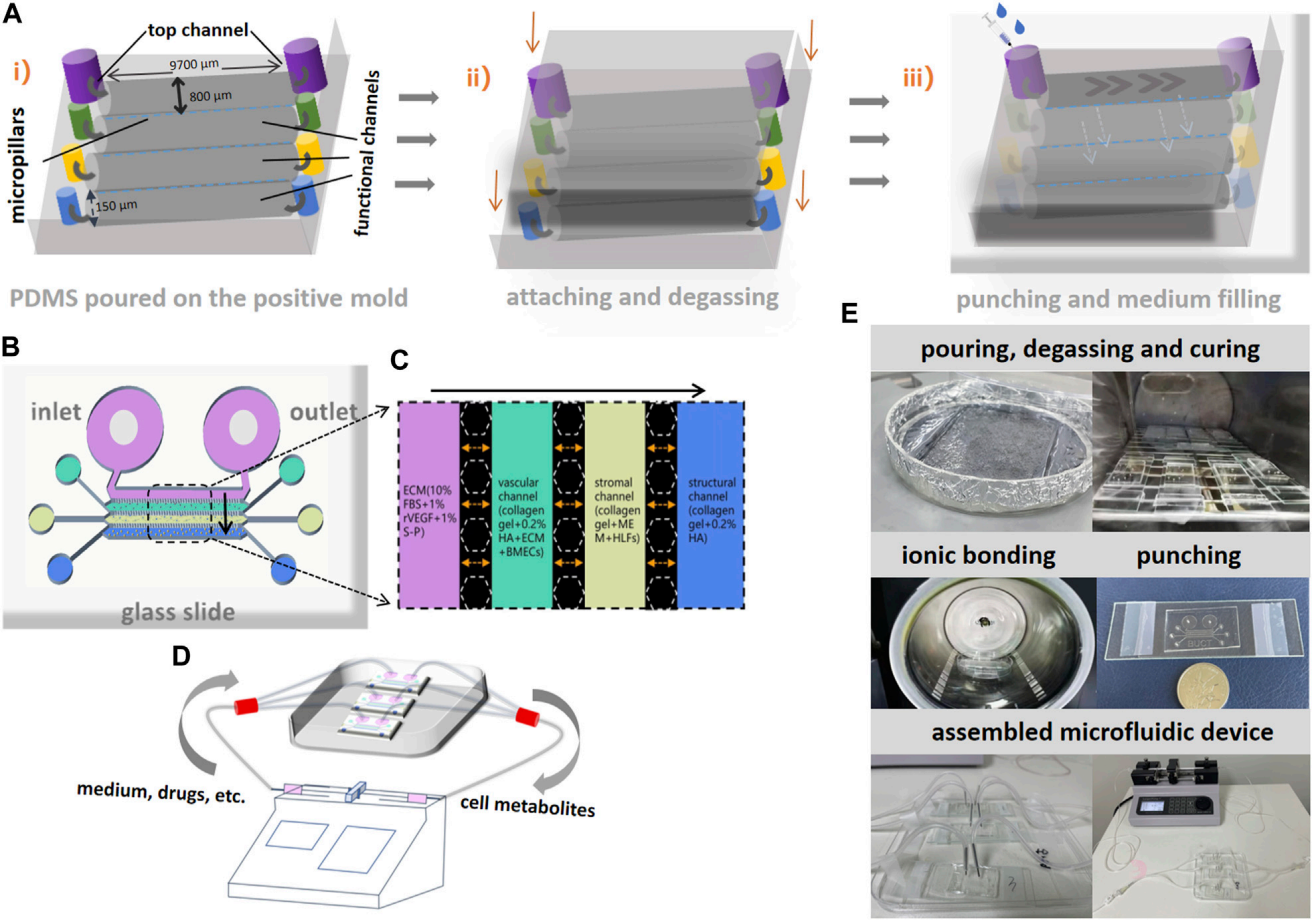
Schematic diagrams of bone-on-a-chip fabrication and assembly of microfluidic devices. **(A)** i), silane treatment was performed after the liquid polydimethylsiloxane (PDMS) was poured on the positive mold to establish the basic structure on bone-on-a-chip. ii), attaching the glass above and degassing after complete curing. iii), a hole was punched at the inlet and outlet of each channel and medium was added through the inlet at the top channel and diffused through micropillars. **(B)**, the schematic diagram of bone-on-a-chip after fabrication. **(C)**, the illustration of different cell mixtures in four channels on the bone-on-a-chip. **(D)**, the schematic diagram of microfluidics and the assembled devices for interventions. The gray arrows indicate the directions of flow inside the device. **(E)**, the brief procedures of chip fabrication and device assembly.

The schematic diagrams of chip fabrications and basic parameters of the chips are presented in Figure 3A. The height of the channels and micropillars is 150 μm. The length of each perfusable channel is 9,700 μm and the width is 800 μm. The gap between two micropillars is 100 μm. Each bone-on-a-chip consists of four channels from top down. The top channel is specifically designed for microfluidics control and medium preservation to mimic the process of metabolites exchange inside the bone microenvironment. In order to elucidate the contents in functional channles clearly, the illustrations of the cell mixture in each functional channel inside the chip are presented in Figures 3B, C. The inlet and outlet of the top channel were connected to two syringe pumps to accelerate the microcirculations inside the microfluidics device after cell seeding. The schematic diagram of the assembled device is shown in Figure 3D. To avoid washing away the incubated cells during delivering nutrients, the infusion of medium was initiated after the complete curing of the mixed gel. The infusion of GC or DNA aptamers, dissolved in endothelial cell medium (ECM) was pumped into the top channel in a steady speed (10 μl/h). Effluent from the outlet was eventually collected into a syringe. The brief procedures of chip fabrication and device assembly are shown in Figure 3E.

### 3.3 Establishment of multi-component model in microfluidic system

**FIGURE 4:**
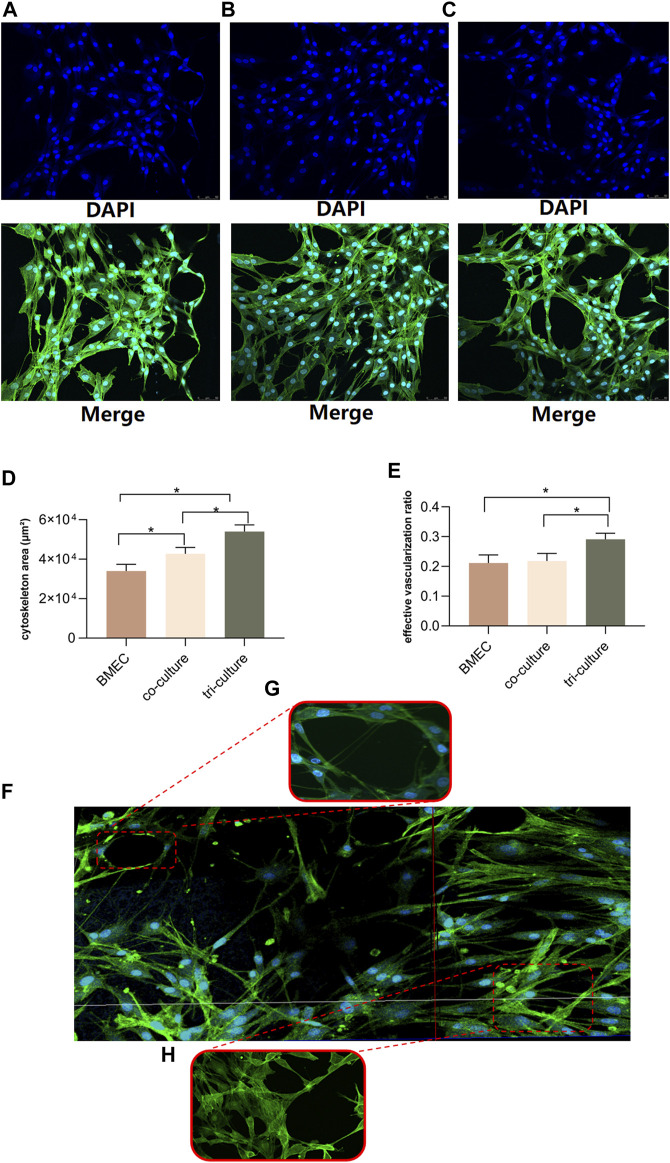
Evaluations for three patterns of cell culture on bone-on-a-chip. **(A–C)**, confocal microscope images (20-fold lens) of the control group, two-component group and multi-component group, respectively. **(D)**, results of the cytoskeleton area in three patterns (n = 3, each group consisted of three repeated chips for analysis) **(E)**, results of the effective vascularization ratio in three patterns (n = 3, each group consisted of three repeated chips for analysis). **(F)**, the reconstructed panorama (10-fold lens) of the vascular channel after the seeding of cell mixture. **(G)**, illustration of the formation of microvessel inside the network (40-fold lens). **(H)**, illustration of the cytoskeleton constructed by the inter-connected microfilaments (40-fold lens). *p < 0.05. Error bars indicate SD. For multiple comparisons between groups, one-way repeated-measures ANOVA was used, followed by the LSD multiple comparison test. ANOVA, analysis of variance.

The confocal microscope (LEICA Microsystem, Germany) was used to evaluate the cytoskeleton growth and angiogenesis status in different culture patterns within bone-on-a-chip. All three culture patterns (BMECs group, two-component group and multi-component group) were evaluated after 3 days of culture (Figures 4A–C). To keep the cells in their expected channels, the microfusion pump would not be initiated until the gel mixture achieved complete curing. The parameters we analyzed among three groups are cytoskeleton area and effective vascularization ratio. The effective vascularization ratio was measured by dividing the microvessel area by the entire cytoskeleton area. Compared to the control and two-component group, the multi-component group displayed more inter-connected microfilaments among the BMECs. According to the statistical analyses, the multi-component group showed the largest coverage of cytoskeleton area and highest effective vascularization ratio (Figures 4D, E). We also found that the structures of microfilaments and microvessels would start to regress in the two-component group after 5 days of culture. However, these structures could still maintain intact after 5 days of culture in the multi-component group. To illustrate the measured objects more vividly, the panorama of the vascular channel, the formation of microvessel and the cytoskeleton constructed by inter-connected microfilaments are demonstrated in Figures 4F–H, respectively.

### 3.4 Docking results to select the optimal modified VR11

**FIGURE 5:**
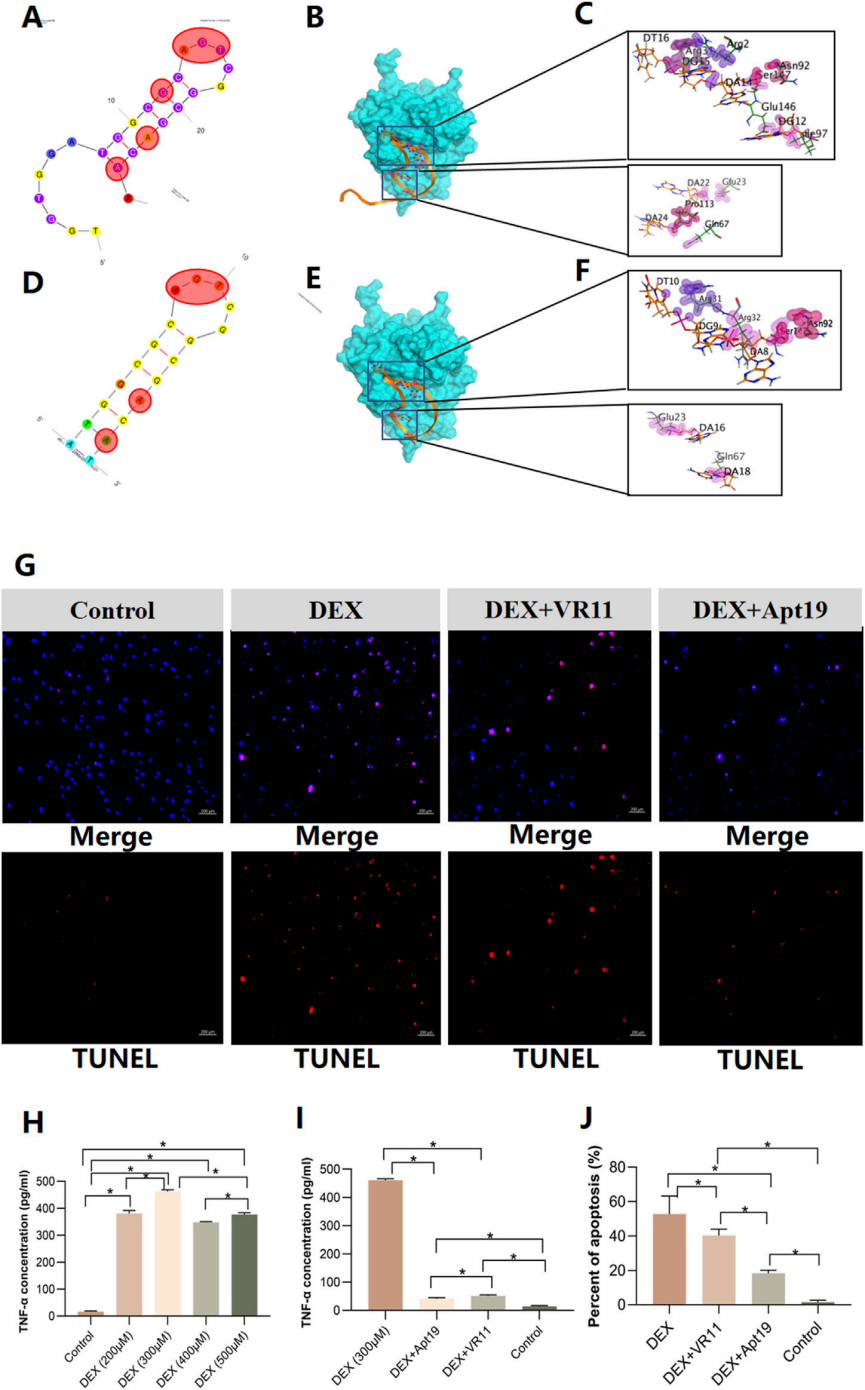
Model simulations of aptamers docking with TNF-α and results of cell apoptosis dectection. **(A–C)**, the secondary structure of VR11 and its nucleotides residues involved in protein-aptamer interaction. The bases in the red shadow region stand for the binding nucleotides. **(D–F)**, the mutated structure of Apt19 and its nucleotides residues involved in protein-aptamer interaction. The bases in the red shadow region stand for the binding nucleotides. **(G)**, images of TUNEL assay for the control group, DEX group, DEX+VR11 group and DEX+Apt19 group, respectively. **(H,I)**, the results of ‘off-chip’ analysis of TNF-α concentrations in different groups measured by the ELISA (n = 6, each group consisted of six repeated wells). **(J)**, the percent of cell apoptosis detected by the TUNEL assay (n = 3, each group consisted of three repeated chips for analysis). For figure **(H–J)**, the error bars indicate SD. For multiple comparisons between groups, one-way repeated-measures ANOVA was used, followed by the LSD multiple comparison test. *p < 0.05. TNF-α, tumor necrosis factor-alpha. DEX, dexamethasone. ELISA, the enzyme-linked immunosorbent assay. ANOVA, analysis of variance.

We first analyzed the original structure, binding energy and residues involved in protein-aptamer interactions of VR11 when binding to TNF-α (Figures 5A–C and Supplementary Table S3). The stem-loop structure of aptamer is viewed as an important part of maintaining its recognition ability or affinity. Therefore, stem-loop structure should be preserved when aptamer is modified. The docking results showed that the first 7 bases of the VR11 had little effect on molecular binding. So we applied truncation procedure of VR11 while preserving its stem-loop structure by truncating the first 6 bases. Meanwhile, to obtain optimal aptamer with more stable structure, we mutated of last base from A to T, i.e., pairing the 7th and 25th bases at the tail (Figure 5D). After the process of truncation and mutation, the sequence of modified VR11 (Apt19) is 5′-ATG​GCG​CAG​TCG​GCG​ACA​T-3’. Subsequently, we analyzed the binding sites and binding energy of Apt19 using the MOE 2020 (CCG Inc, Canada). According to the docking results, the Apt19 can still retain most of the original action sites while decreasing the binding energy simultaneously (Figures 5E, F and Supplementary Table S4). In addition, the stability of the sample would be higher because of the complementary double-stranded structure at the tail of Apt19.

On the other hand, given the superiority of Apt19, we also performed the docking procedure of the dimerization of Apt19. The sequence of the dimer-Apt19 is 5′-ATG​GCG​CAG​TCG​GCG​ACA​TTT​TTT​TTT​TTA​TGG​CGC​AGT​CGG​CGA​CAT-3’. The linker consisted of 10 nt of thymine in the middle of the sequence. However, given that the predicted model is rigid docking, the bivalent aptamer obtained from dimerization revealed no flexibility. Therefore, it was difficult to maintain the original binding sites to achieve the optimal affinity. The altered binding sites of interaction also indicate less possibility of better affinity after the dimerization (Supplementary Figure S1). In summary, the truncated aptamer (Apt19) is more likely to be stable in its second structure while maintaining the binding sites with TNF-α. Hence, we selected the truncated aptamer (Apt19) for further applications in the bone-on-a-chip.

### 3.5 Up-regulation of TNF-α in bone-on-a-chip treated with GCs

The collections of effluent from the chips were detected for TNF-α concentrations. Mutual exchanges of metabolites were permitted through gaps between the linear micropillar arrays. TNF-α absorbance was measured at 450 nm and 630 nm and quantified by the linear regression based on the standard curve. First, the concentrations of TNF-α in the chips treated with dexamethasone (DEX) were detected. Five groups were analyzed: control group and DEX groups (200μM, 300μM, 400μM, 500μM, respectively). The cell metabolites were collected after 24-h DEX treatment for the enzyme-linked immunosorbent assay (ELISA). According to the results, all DEX groups showed significant increase in TNF-α concentrations compared with that in the control group. Among four DEX groups, the 300 μM group revealed highest TNF-α concentration after DEX treatmentin the bone-on-a-chip (Figure 5H). Furthermore, we quantified the TNF-α concentrations in bone chips after 24-h treatments of the DEX alone (300 μM), DEX (300 μM)+VR11 (2 μM), DEX (300 μM)+Apt19 (2 μM) and control group. Results from the ELISA showed that the TNF-α concentrations in both the DEX+VR11 and DEX+Apt19 groups were significantly lower than that in the DEX group (Figure 5I). Additionally, there was a statistical significance in TNF-α concentration between the DEX+VR11 group (48.38 pg/ml) and DEX+Apt19 group (39.50 pg/ml).

### 3.6 DNA aptamers attenuated cell apoptosis of BMECs treated with GCs

Using the DNA aptamers, we next investigated whether the aptamers could inhibit the apoptosis of BMECs treated with GCs in the microfluidic system. Four groups were investigated by the TUNEL assay: DEX (300 μM) group, DEX (300 μM)+VR11 (2 μM) group, DEX (300 μM)+Apt19 (2 μM) group and control group. As shown in the Figure 5G, compare with the BMECs treated with DEX alone, the cells treated with DEX and aptamers were demonstrated to trigger less apoptosis under the same DEX concentration in bone-on-a-chip. To compare the protective effects between the Apt19 and VR11, the optimal concentration of both aptamers (VR11 and Apt19) was selected as previously reported (Orava et al., 2013). Accoring to the TUNEL results (Figure 5J), the cells in the DEX+Apt19 group were found to demonstrate the least percent of apoptosis among four groups. Besides, the BMECs in the DEX+Apt19 group showed significantly less apoptosis than those in DEX+VR11 group.

### 3.7 DNA aptamers alleviated damages to cytoskeleton and angiogenesis by GCs

**FIGURE 6:**
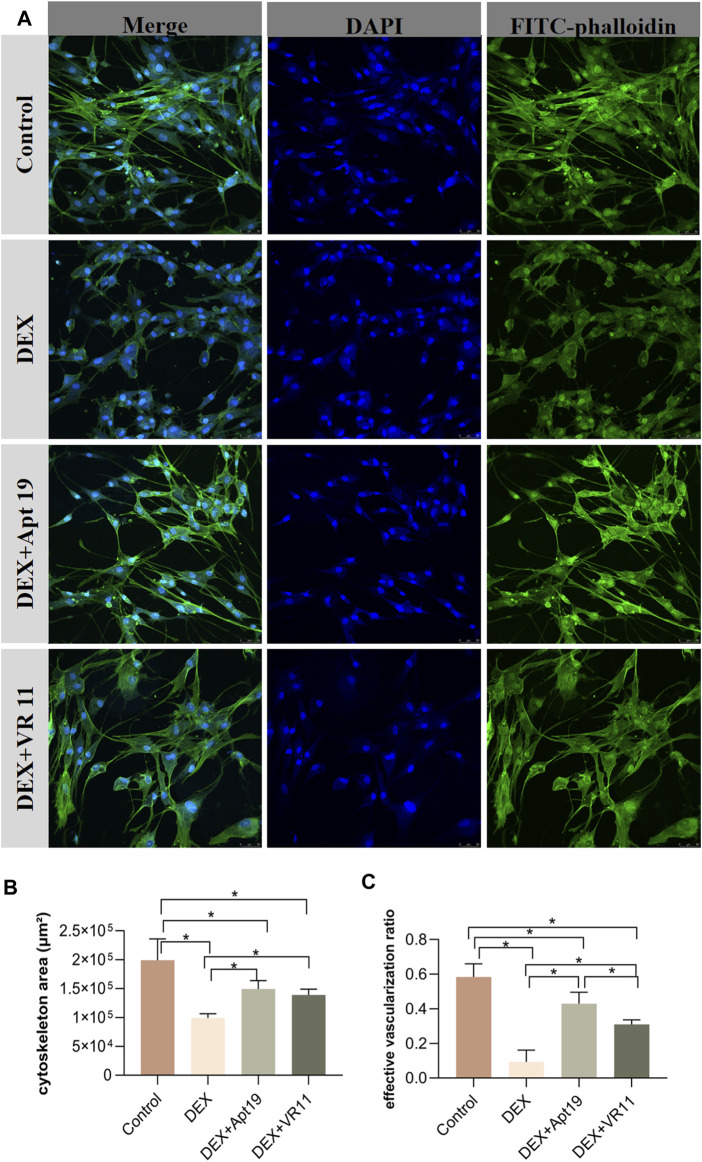
The protective effects of DNA aptamers on cytoskeleton and angiogenesis. **(A)**, the confocal microscope images (20-fold lens) of the control group, DEX group, DEX+Apt19 group and DEX+VR11 group, respectively. **(B,C)**, the statistical results of the cytoskeleton area and the effective vascularization ratio in all four groups (n = 3, each group consisted of three repeated chips for analysis). *p < 0.05. Error bars indicate SD. For multiple comparisons between groups, one-way repeated-measures ANOVA was used, followed by the LSD multiple comparison test. DEX, dexamethasone. ANOVA, analysis of variance.

Similar groups were designed for the evaluations of cytoskeleton area and angiogenesis status: DEX (300 μM) group, DEX (300 μM)+VR11 (2 μM) group, DEX (300 μM)+Apt19 (2 μM) group and control group. All chips were stained with DAPI (364 nm) and FITC-phalloidin (496 nm) for confocal images. As shown in the Figure 6A, the DEX group showed obviously retrogressive structures of microfilaments and inhibited angiogenesis compared with the control group. Interestingly, the degenerations of the cytoskeleton were alleviated when we treated the cells with aptamers and DEX. The inhibitory effect of DEX on angiogenesis within bone-on-a-chip also became weaker in the DEX+Apt19 and DEX+VR11 groups. The effective vascularization ratio in DEX+Apt19 group was found to be significantly higher than that in DEX+VR11 group, while no statistical significance was found between two groups in terms of cyoskeleton area. Both aptamer groups demonstrated less cytoskeleton area and angiogenesis compared with the control group (Figures 6B, C).

## 4 Discussion

For the first time, we present a microfluidic device of bone-on-a-chip to study the therapeutic effect of DNA aptamers on ONFH model based on the human-derived cells. Compared with previous bone chips (Chou et al., 2020; Glaser et al., 2022; Jusoh et al., 2015), we created a microfluidic bone microenvironment that mimics the microcirculation inside human bone tissue to achieve the ‘off-chip’ analysis of cell metabolites within the microenvironment. Besides, our bone-on-a-chip consisted of a group of human-derived cells. We designed the multi-component culture of BMECs, HLFs and HA to stand for the compositions of the endothelial cells, the stromal cells and inorganic substances in bone tissue, respectively. Also, the microfluidic control within bone-on-a-chip not only provided essential nutrients to the functional channels, but also offered us an opportunity for further explorations of intercellular communications after interventions.

To demonstrate the process of vascularization in bone-on-a-chip, we used the ratio of microvessel area to the cytoskeleton area as the effective vascularization ratio to evaluate the status of angiogenesis. The application of this parameter was inspired and referred to the previous studies (Sano et al., 2018; Whisler et al., 2014). Whisler et al. gave a definition of the ‘effective diameter’, calculated as the ratio of vascularized area to total length of engineered microvascular networks (Whisler et al., 2014). In our current study, the cytoskeleton area was calculated as the whole engineered microvascular networks. While the effective vascularization ratio was calculated to evaluate the formations of capillary-like microvessels inside the networks. According to the results, the multi-component group showed the largest coverage of cytoskeleton area and highest effective vascularization ratio. This demonstration indicated that the multi-component model revealed the more stable cell morphologies and tight microvascular structures. Unique from other bone chips (Chou et al., 2020; Glaser et al., 2022; Jusoh et al., 2015), our multi-component model was established on a group of human-derived endothelial cells and stromal cells because a single cell line used in preclinical validations frequently did not fully reflect the physiology and complexity in humans. We also added HA to the multi-component compositions to simulate the proportion of mineral substances in bone tissues. Although previous reports indicated that the two-component culture of HA and BMECs could enhance the cell growth (Li et al., 2021), the effects on cell growth and angiogenesis status in multi-component culture pattern still remains unclear. Our design of three parallel culture patterns was to make sure that each group must be cultivated under the same circumstance. Results showed that multi-component group exhibited the highest effective vascularization ratio in three groups, while there was no significant difference between the two-component group and control group. One possible explanation was that the existence of fibroblasts in the multi-component model helped to stabilize the newly-formed microvascular networks and prevent the networks from early regression (Jeon et al., 2014; Whisler et al., 2014). Therefore, the importance of our multi-component model may lie in the attempt to mimic physiological cell-matrix interface and maximize the interactions between endotelial cells and stromal support cells.

TNF-α is expressed widely in endothelial cells as both transmembrane and in soluble forms (Galvão et al., 2013). TNF-α-induced cell death has been demonstrated as a critical role in the developments of various diseases in multiple systems (Brenner et al., 2015; Gunther et al., 2011; Roca et al., 2019), including early-stage development of ONFH (Zheng et al., 2020). In this study, the significant increase of TNF-α concentration in the clinical specimens of ONFH patients and in the microfluidic system after DEX treatment confirmed the underlying relations between TNF-α and GC-induced ONFH. This ‘off-chip’ analytic strategy offers us the possibility of real-time tracking and analysis of metabolites within the microfluidics in the near future.

The idea of using the antagonists against TNF-α to block its cascade activities was initially converted into practice in 1994 for patients with rheumatoid arthritis (Elliott et al., 1994). As an antidote for specific receptor, the DNA aptamer (a single-stranded oligonucleotide) can be artificially designed and chemically synthesized without strict storage conditions. Additionally, aptamers are capable of overcoming some major problems with most of the current TNF blockers, such as immune reactions, immunosuppressions, increased risk of serious infections and high costs (Menegatti et al., 2019; Croft and Siegel, 2017). Our investigations showed that less necrotic BMECs were observed in the bone chips with lower concentrations of TNF-α. Similarly, better cytoskeleton growth and angiogenesis status were discoverd in the chips with lower concentrations of TNF-α. We also found that the process of angiogenesis was restored to a greater extent in the DEX+Apt19 group compared with that in the DEX+VR11 group. One possibly explanation is that Apt19 is likely to generate more protein-aptamer interactions because of its lower binding energy and complementary double-stranded structure so as to block the activities of TNF-α more efficiently. We also believe that the process of angiogenesis should be based on formations of the cytoskeleton. In other words, the impairments of the vascularization were supposed to occur prior to the deteriorations of the cytoskeleton after the treatment with DEX. This explanation might be helpful to understand the reason why there was a statistcal significance in the effective vascularization ratio, rather than the cytoskeleton area, between the DEX+Apt19 and DEX+VR11 groups.

There are several limitations in this study. First, we did not take the level of tissue degradation into consideration when performing the histological analysis. Second, the multi-component ONFH model still lacks of bone-related cells (e.g., bone marrow stromal cells, etc) to study crosstalks with BMECs. Finally, there was no further *in vivo* studies or animal models to justify the therapeutic effects of TNF-α aptamer.

## 5 Conclusion

In summary, we present a multi-component ONFH model based on the microfluidic platform, allowing to mimic physiological cell-matrix interface and predict responses to drug therapies. Unlike previous bone chips, this microfluidic bone-on-a-chip based on isolated functional channels achieved ‘off-chip’ analysis of cell metabolites and consisted of a group of human-derived cells. We explored the idea of using DNA aptamer specifically against TNF-α to block its cascade effects on endothelial cells. Our findings provided initial evidence on the possible potentials of TNF-α aptamer as a new type of TNF-α inhibitor for patients with ONFH. Investigating therapeutic potentials of aptamers implemented in organ-on-a-chip should gain more attention in the future work.

## Data Availability

The datasets concerning human specimens presented in this article are not readily available because of the privacy policy in the informed documents we signed with enrolled patients before the study. Requests to access the datasets of human histological features and analysis should be directed to the corresponding author Wei Sun. The other related datasets in this study have been deposited in the following link: https://www.jianguoyun.com/p/DVcwCVMQ5ObZCxit74oFIAA. The uploaded datasets are available to everyone without accession code.
