# Supplementary material for: Establishment of the multi-component bone-on-a-chip: to explore therapeutic potential of DNA aptamers on endothelial cells
Source: Front Cell Dev Biol. 2023 Jun 12;11:1183163. doi: 10.3389/fcell.2023.1183163 (PMC10291622; doi:10.3389/fcell.2023.1183163)
Supplement: Supplementary file 1 [file Table1.DOCX]

| No. | Diagnosis | Age | Gender | Garden Classification | BMI(kg/m^2^) |
| --- | --- | --- | --- | --- | --- |
| P1 | Femoral neck fracture | 76 | M | IV | 24.22 |
| P2 | Femoral neck fracture | 79 | F | IV | 25.07 |
| P3 | Femoral neck fracture | 80 | F | IV | 23.89 |
| P4 | Femoral neck fracture | 81 | F | IV | 23.19 |
| P5 | Femoral neck fracture | 85 | M | IV | 22.87 |
| P6 | Femoral neck fracture | 75 | F | IV | 25.10 |
| P7 | Femoral neck fracture | 77 | F | IV | 24.89 |
| P8 | Femoral neck fracture | 79 | M | IV | 24.32 |
| P9 | Femoral neck fracture | 78 | M | IV | 24.46 |
| P10 | Femoral neck fracture | 76 | M | IV | 24.18 |

| No. | Diagnosis | Age | Gender | ARCO Stage | BMI(kg/m^2^) |
| --- | --- | --- | --- | --- | --- |
| P1 | ONFH | 50 | F | IIIc | 23.52 |
| P2 | ONFH | 60 | F | IIIc | 24.88 |
| P3 | ONFH | 61 | M | IV | 24.14 |
| P4 | ONFH | 55 | F | IIIc | 25.23 |
| P5 | ONFH | 53 | M | IIIc | 24.78 |
| P6 | ONFH | 49 | F | IIIc | 24.60 |
| P7 | ONFH | 51 | M | IIIc | 23.97 |
| P8 | ONFH | 58 | M | IV | 24.52 |
| P9 | ONFH | 57 | M | IIIc | 23.87 |
| P10 | ONFH | 59 | F | IV | 24.78 |

**Table S1 The deatiled characteristics of all enrolled patients in two groups**
