# Supplementary figures and images for: Establishment of the multi-component bone-on-a-chip: to explore therapeutic potential of DNA aptamers on endothelial cells
Source: Front Cell Dev Biol. 2023 Jun 12;11:1183163. doi: 10.3389/fcell.2023.1183163 (PMC10291622; doi:10.3389/fcell.2023.1183163)

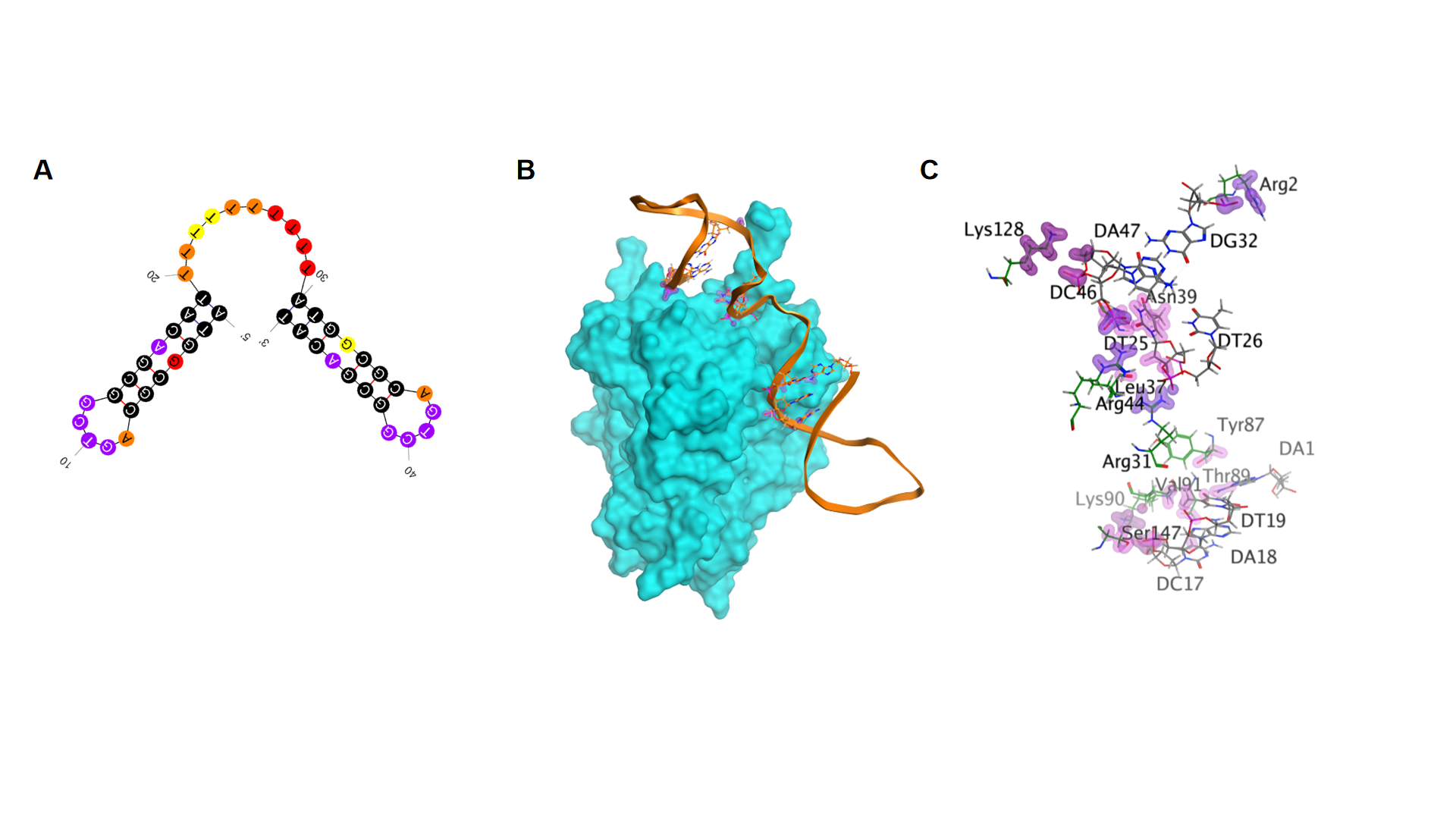

Supplement: Supplementary file 2 [file Image1.TIF]
