# Supplementary material for: Establishment of the multi-component bone-on-a-chip: to explore therapeutic potential of DNA aptamers on endothelial cells
Source: Front Cell Dev Biol. 2023 Jun 12;11:1183163. doi: 10.3389/fcell.2023.1183163 (PMC10291622; doi:10.3389/fcell.2023.1183163)
Supplement: Supplementary file 3 [file Table2.DOCX]

|  | Top channel  (ECM) | Vascular channel  (BMECs/BMECs+0.2% HA) | Stromal channel  (HLFs) | Structural channel  (0.2% HA) |
| --- | --- | --- | --- | --- |
| BMECs group | √ | √(BMECs) |  | √ |
| Two-component group | √ | √(BMECs+0.2% HA) |  | √ |
| Multi-component group | √ | √(BMECs+0.2% HA) | √ | √ |

**Table S2 The deatiled components of three different culture patterns**
