# Supplementary material for: Establishment of the multi-component bone-on-a-chip: to explore therapeutic potential of DNA aptamers on endothelial cells
Source: Front Cell Dev Biol. 2023 Jun 12;11:1183163. doi: 10.3389/fcell.2023.1183163 (PMC10291622; doi:10.3389/fcell.2023.1183163)
Supplement: Supplementary file 4 [file Table3.DOCX]

**Table S3 The contact types, interaction sites and binding energy of VR11 binding to TNF-α**

| **Contact type** | **Aptamer** | **TNF-α** |
| --- | --- | --- |
| DIH | 16 | 31 |
| DH | 14 | 92 |
| D | 22 | 23 |
| D | 12 | 146 |
| DH | 24 | 113 |
| D | 12 | 97 |
| D | 14 | 147 |
| D | 24 | 67 |
| DI | 15 | 2 |
| Binding Energy | -58.4801 kcal/mol |  |

D, displays VdW distance interaction energies. I, displays ionic bond contacts. H, displays hydrogen bond contacts.
