# Supplementary material for: Establishment of the multi-component bone-on-a-chip: to explore therapeutic potential of DNA aptamers on endothelial cells
Source: Front Cell Dev Biol. 2023 Jun 12;11:1183163. doi: 10.3389/fcell.2023.1183163 (PMC10291622; doi:10.3389/fcell.2023.1183163)
Supplement: Supplementary file 5 [file Table4.DOCX]

**Table S4 The contact types, interaction sites and binding energy of Apt19 binding to TNF-α**

| **Contact type** | **Aptamer** | **TNF-α** |
| --- | --- | --- |
| DH | 8 | 92 |
| DI | 10 | 31 |
| D | 18 | 67 |
| D | 16 | 23 |
| D | 8 | 147 |
| D | 9 | 32 |
| Binding Energy | -60.1290 kcal/mol |  |

D, displays VdW distance interaction energies. I, displays ionic bond contacts. H, displays hydrogen bond contacts.
